# Visual System Impairment in a Mouse Model of Krabbe Disease: The Twitcher Mouse

**DOI:** 10.3390/biom11010007

**Published:** 2020-12-23

**Authors:** Ilaria Tonazzini, Chiara Cerri, Ambra Del Grosso, Sara Antonini, Manuela Allegra, Matteo Caleo, Marco Cecchini

**Affiliations:** 1 NEST, Istituto Nanoscienze-CNR and Scuola Normale Superiore, Piazza San Silvestro 12, 56127 Pisa, Italy; ilaria.tonazzini@sns.it (I.T.); ambra.delgrosso@sns.it (A.D.G.); sara.antonini@gmail.com (S.A.); 2 Istituto Neuroscienze-CNR, Via G. Moruzzi 1, 56124 Pisa, Italy; chiara.cerri@unipi.it (C.C.); manuela.allegra84@gmail.com (M.A.); matteo.caleo@unipd.it (M.C.); 3 Department of Pharmacy, University of Pisa, Via Bonanno Pisano 6, 56126 Pisa, Italy; 4 Department of Neuroscience, Institut Pasteur, 25 Rue du Dr Roux, 75015 Paris, France; 5 Department of Biomedical Sciences, University of Padua, Viale G. Colombo 3, 35131 Padua, Italy

**Keywords:** Krabbe disease, Twitcher mouse, psychosine, visual system, visual cortex, astrogliosis

## Abstract

Krabbe disease (KD, or globoid cell leukodystrophy; OMIM #245200) is an inherited neurodegenerative condition belonging to the class of the lysosomal storage disorders. It is caused by genetic alterations in the gene encoding for the enzyme galactosylceramidase, which is responsible for cleaving the glycosydic linkage of galatosylsphingosine (psychosine or PSY), a highly cytotoxic molecule. Here, we describe morphological and functional alterations in the visual system of the Twitcher (TWI) mouse, the most used animal model of Krabbe disease. We report in vivo electrophysiological recordings showing defective basic functional properties of the TWI primary visual cortex. In particular, we demonstrate a reduced visual acuity and contrast sensitivity, and a delayed visual response. Specific neuropathological alterations are present in the TWI visual cortex, with reduced myelination, increased astrogliosis and microglia activation, and around the whole brain. Finally, we quantify PSY content in the brain and optic nerves by high-pressure liquid chromatography-mass spectrometry methods. An increasing PSY accumulation with time, the characteristic hallmark of KD, is found in both districts. These results represent the first complete characterization of the TWI visual system. Our data set a baseline for an easy testing of potential therapies for this district, which is also dramatically affected in KD patients.

## 1. Introduction

Krabbe disease (KD, or globoid cell leukodystrophy; OMIM #245200) is an inherited neurodegenerative condition belonging to the class of the lysosomal storage disorders (LSDs). It is caused by multiple genetic alterations in the gene encoding for the enzyme galactosylceramidase (GALC; EC 3.2.1.46) or, in some rare cases, for the Sphingolipid activator protein saposin A (SapA) [1]. The deficiency of GALC or SapA does not allow the proper functioning of the complex sphingolipid cell pathway, which is especially crucial for myelinating cells [2]. More specifically, the GALC-SapA complex is also responsible for cleaving the glycosydic linkage of galatosylsphingosine (psychosine or PSY), a highly cytotoxic molecule which is able to insert into the cell membrane, disrupt raft architecture, and presumably deregulate multiple cell signaling cascades [3,4]. Still, the exact mechanism through which this deadly molecule exerts its cytotoxicity has not been completely elucidated yet [5], even if the PSY hypothesis has been recently confirmed [6]. The most proved explanations about KD molecular pathogenesis sustain that PSY primarily causes a massive death of oligodendrocytes and Schwann cells with consequent demyelination of central and peripheral nervous system (CNS and PNS), which is followed by neurodegeneration owing to the disruption of the neuronal-glial homeostasis [7]. Widespread cell death also leads to activation of the inflammatory cascade, which recruits macrophages and activates microglia, amplifying the production of cytotoxic molecules [8]. The inflammatory response to demyelination is then followed by astrocytic gliosis [9]. In the areas of active demyelination, furthermore, multinucleated macrophages, called globoid cells, are often clustered around blood vessels [10,11]. The only available treatment for presymptomatic human patients is hematopoietic stem cell transplantation (HSCT) [10], which in a few cases could delay the symptoms onset and progression. However, at the moment no effective cure exists for KD. Available options are symptomatic and supportive only [12]. The first hallmarks of the disease are generally alteration of neuronal conduction and neurological dysfunctions. Four different clinical forms of KD are usually described according to symptom onset: infantile, late infantile, juvenile, and adult. The infantile phenotype (95% of the known cases) has usually onset within first 6 months of life and leads to death by age of 2–4 years [13,14,15]. Hyperirritability, hypersensitivity to the external environment, stiffness of the limbs, and episodic fever of unknown origin are firstly reported. The psychomotor functions deteriorate quickly with marked hypertonicity, and the backward bent head. At the latest stage of the disease, the infants are in a decerebrate posture and become blind and unresponsive of their surroundings. The late infantile form, instead, has onset between 6 months to 3 years. Irritability, psychomotor regression, stiffness, ataxia, and loss of vision are frequent initial symptoms. The course is progressive and results in death approximately 2 or 3 years after the onset. The juvenile form presents onset between 3 and 8 years. Patients commonly develop loss of vision, hemiparesis, ataxia, and psychomotor regression [16,17]. Adult form, whose onset is after 20 years, has a milder phenotype and a slower rate of progression, allowing sometimes a normal life span to patients. Some individuals remain stable for long periods of time, while others show a steady decline in a vegetative state and death [18]. Patients usually experience a progressive decline of their visual skills culminating with blindness.

An anatomical characteristic of KD patients visual system is an extensive demyelination of the optic nerve [19] that coexists with an early stage optic nerve enlargement due to the presence of numerous globoid cells [20,21]. Demyelination was also found in the white matter of postmortem KD brain that additionally displayed a massive gliosis [19]. Beside the general white matter volume loss in the brain of children with KD disease, alterations of visual system in KD patients have been reported in the clinic also in terms of functionality. Indeed, electroencephalographic recordings upon visual stimulation showed abnormal visual evoked potential (VEP) response in KD patients [15,22]. 

As evident, common features of all clinical KD forms are defects of the visual system. However, although visual system impairment is a significant hallmark of KD, it has not yet received much attention from the scientific community, and has not yet been sufficiently characterized. For example, a detailed analysis of which visual response parameters are compromised in KD is still lacking and almost nothing has been investigated about the visual system in the most used mouse model, the Twitcher mouse (TWI). The TWI mouse presents an autosomal recessive mutation in the GALC gene with no counterpart in humans [23], and pathological phenotype closely recapitulates human pathology [24,25]. TWI mice are the most used animal model for the testing of experimental therapies for KD, such as enzyme replacement therapies or gene therapy [26,27]. With the disease progression animals experience severe tremors, paralysis of hind legs and neck muscles, important weight loss, and demyelization, and lifespan rarely extends beyond PND40 [9,23]. Rather than for KD, visual defects have been broadly studied and found in animal models of other LSDs, such as the Cystinosis (CTSN) [28,29], Sandhoff and GM1 gangliosidosis [30], and Mucopolysaccharidosis type IIIA [31].

In the present study, for the first time, we investigate the visual system of the TWI mouse. We report in vivo electrophysiological recordings of the basic functional properties (acuity, contrast sensitivity, and visual evoked potentials latency) of the primary visual cortex of TWI mice (PND 30-36). In parallel, we investigate the presence of neuropathological alterations in TWI CNS tissues, as possible biomarkers of TWI condition, and quantify the PSY content in brain and optic nerves.

## 2. Materials and Methods

### 2.1. Animals

Twitcher heterozygous mice (TWI^+/−^ C57BL6 mice; Jackson Labs), kindly donated by Dr. A. Biffi (San Raffaele Telethon Institute for Gene Therapy, Milan, Italy), were used as breeder pairs to generate homozygous TWI^−/−^ mice (TWI). Animals were maintained under standard housing conditions and used according to the protocols and ethical guidelines approved by the Ministry of Health (Permit Numbers: CBS-not. 0517, approved the 04/01/2018; 535/2018-PR, approved 09/07/2018). For genotyping purpose, mice genomic DNA was extracted from clipped tails by Proteinase K digestion and subsequent genomic DNA extraction (EUROGOLD Tissue-DNA Mini Kit, EuroClone), as previously reported. The genetic status of each mouse was later determined from the genome analysis of the TWI mutation, as reported from [9,32]. TWI and WT (TWI^+/+^) littermate animals at postnatal day (PND) between PND20 and PND43 were used for experiments, while the heterozygous (TWI^+/−^) littermates were retained for the colony maintenance. 

### 2.2. Visual Cortex Recording

Electrophysiological recordings were performed as described previously [33,34]. Mice were anesthetized with Hypnorm/Hypnovel (in water; 0.3 mL/20 g; VetaPharma, Leeds, UK) and placed in a stereotaxic apparatus. A portion of the skull overlying the binocular visual cortex was drilled on one side. A tungsten electrode (1 MΩ; FHC, Bowdoin, ME, USA) was mounted on a three-axis motorized micromanipulator (Scientifica, Uckfield East Sussex, UK) and inserted into the binocular portion of the visual cortex (approximately 2.9 mm lateral from midline and in correspondence with lambda in PND30-36 mice). VEPs were recorded from 3 to 4 penetrations/animal and the electrode was positioned at 100 and 400 μm depth within the cortex. Electrical signals were amplified (10,000-fold), band-pass filtered (0.3–100 Hz), digitized, and averaged in synchrony with the stimulus contrast reversal (temporal frequency, 1 Hz). Analysis of the amplitude of VEP responses was performed blind to animal genotype. Visual stimuli were gratings of various spatial frequencies and contrast generated by a VSG2/5 card (Cambridge Research Systems, Rochester, UK) on a display (Sony Multiscan G500; Sony Europe B.V., Milano, Italy; mean luminance, 15 cd/m^2^) that was positioned 20–30 cm in front of the mouse eyes to include the central visual field (110 × 85° of visual angle).

The visual response was measured as the peak to through amplitude (µV). We also collected the responses to 0% contrast (blank stimuli) to measure noise level. Visual acuity (c/deg) was assessed after presentation of gratings of variable spatial frequencies (90% contrast). Contrast threshold was determined via the presentation of gratings of different contrasts and fixed spatial frequency (0.06 c/deg). Visual acuity and contrast threshold were determined as the highest spatial frequency and lowest contrast that evoked a VEP response greater than the mean value of the noise. We used *n* = 7 TWI and *n* = 6 WT mice, age range PND30-36.

### 2.3. Immunohistochemistry and Confocal Imaging

TWI and WT mice (*n* = 4 for each genotype, PND21-36) were deeply anesthetized and perfused transcardially with phosphate buffer saline (PBS) and subsequently with 4% paraformaldehyde (PFA). After perfusion, the brains were stored at 4 °C in a 4% PFA solution for minimum 2 days. Consecutive coronal sections of the visual cortex, 50 μm thick, were cut with a microtome (VT 1000 S, Leica BIOSYSTEM, Buccinasco (MI), Italy) Sections were maintained at 4 °C in PBS until use. For the immunohistochemistry staining [35], sections were transferred in a 24 well cell culture plate (maximum of 3 sections per well) and incubated with blocking solution (3% bovine serum albumin, 0.3% Triton X-100 in PBS; 1 mL per well) for 1 h at room temperature (RT). Then, the blocking solution was removed and the primary antibody mix (1% BSA, 0.3% Triton X-100, antibody at the optimal dilution and PBS to reach 1 mL) was added to the well and left overnight at 4° under gentle shaking. We used the following antibodies: anti-GFAP (ab7260 Abcam, 1:1000), anti-MBP (ab980 Merck Millipore, 1:500), and anti-Iba-1 (Wako 019/1974, 1:500). The next morning, primary antibody solution was removed and 3 washes with 1 mL of PBS were made (10 min each). Then, secondary antibody solution was added (BSA 1%, Triton X-100 0.3%, antimouse, or antirabbit Alexa 647 1:1000, antimouse or anti-rabbit Alexa 488 1:000 and PBS to reach 1 mL total volume). After 2 h of incubation at RT, other 3 washes with PBS were made. Sections were then stained with a Hoechst solution for 1 min and then mounted on SUPERFROST Microscope Slides (Thermo Scientific Waltham, MA, USA) with Vectashield Antifade Mounting Medium (VECTOR LABORATORIES), also with DAPI. Finally, slides were sealed and stored at 4 °C until confocal imaging.

Cortical sections were examined with a Leica Confocal microscope, Buccinasco (MI), Italy using a 10× air objective. For each section, confocal series of a step size of 2 μm were obtained throughout the whole section thickness (50 μm) and collapsed as an average. Acquisition parameters of each labeling (width along the z-axes, laser intensity, photomultiplier gain, and pinhole size) were kept constant for both experimental groups (TWI and WT mice).

### 2.4. Western Blot

Western blot analysis on brain lysates was performed to assess the levels of MBP, GFAP, and Iba-1 proteins, such as biomarkers of demyelination, astrogliosis, and microglia-macrophages activation, respectively. For mouse tissues, the extracted brains (without olfactory bulbs and cerebellum) were lysed on ice in RIPA buffer (Merck KGaA, Darmstadt, Germany R0278) containing protease and phosphatase inhibitors cocktail (cOmplete and PhosSTOP, Roche Diagnostics, Basel, Switzerland). Brain lysates were centrifuged (15,000× *g* for 25 min, 4 °C), and then, the supernatants were tested for protein concentration by a protein assay kit (Micro BCA™, Thermo Scientific Pierce). The samples were mixed with Laemmli buffer containing β-mercapto-ethanol (5% final concentration), boiled for 5 min, and used for gel electrophoresis (or kept at −80 °C). Brain lysates (60 µg/lane) were processed by immunoblot, as in [26,36]. Briefly, samples were resolved by gel electrophoresis (SDS-PAGE) using Gel Criterion XT-Precast polyacrylamide gel 4–12% Bis-Tris (Bio-Rad, Hercules, CA, USA), transferred to nitrocellulose membranes, and probed overnight at 4 °C with primary antibodies. We used the following antibodies against: GFAP (1:1000; Synaptic Systems #173211), MBP (1:1000; Abcam, ab62631), Iba-1 (1:1000; Sigma, MABN92), Tubulin (1:2000; Merck, T6074), and GAPDH (1:3000; Merck, G8795). Membranes, after incubation with the appropriate peroxidase-linked secondary antibodies (goat anti-Rabbit/Mouse IgG-HRP Conjugate, Bio-Rad; 1:2500), were developed by the ClarityTM (Bio-Rad, 170-5060) enhanced chemiluminescent (ECL) substrates. The chemiluminescent signal was acquired by ImageQuant LAS400 scanner (GE Healthcare Life Sciences, Uppsala, Sweden). The density of immunoreactive bands was quantified by ImageJ; the results were normalized to the relative total GAPDH or Tubulin protein levels (according to the more appropriate molecular weight) and reported in percentage with respect to WT levels. At least 18 mice were analyzed for each genotype; age range was PND29-43 for WT mice, and PND30-40 for TWI mice.

### 2.5. Psychosine and GALC Activity Quantification in TWI Tissues

Brain and optic nerve lysates, prepared as above, were used to measure the content of psychosine (PSY) in TWI mice, as in [5].

For lipid extraction, a mixture was prepared combining each lysate, N,N-dimethylsphingosine 1.250 μM (N,N-DMS, the selected internal standard for LC/MS-MS; SML0311-5MG; Merck KGaA, Darmstadt, Germany and Milli-Q water. Then, a chloroform/methanol solution (2:1) was added. Samples were vortexed, left at RT for 10 min, and supplemented with NaCl 0.9% *w*/*v* in Milli-Q water. The biphasic mixture was centrifuged at 800 g for 30 min, and the lower layer was collected and evaporated to dryness under vacuum at 30 °C. The residue was dissolved in 50 μL of methanol/formic acid 100/0.1 and processed for HPLC/MS. 

HPLC/MS quantitation was performed on a Shimadzu Nexera UHPLC chromatograph (Shimadzu Europa GmbH, Duisburg, Germany) interfaced with an AB Sciex 3200 mass spectrometer (AB SCIEX). HPLC analyses were performed on a Vydac C4 1  ×  250 mm column (particle size 4 μm), using water/methanol/isopropanol/formic acid 40/55/5/0.1 (A) and methanol/isopropanol/formic acid 95/5/0.1 (B) as mobile phases at 0.1 mL/min flow. Runs were performed under a 45 min linear gradient from 0% to 100% of solvent B, followed by a 5 min purge step at 100% of B and by a 10-min re-equilibration step to the starting conditions. MRM analyses were performed under the following conditions: ion spray voltage: 5000 V, source temperature 350 °C, declustering potential 50 V, collision energy variable (see value in parentheses), ion source gas 20 L/min, curtain gas: 25 L/min. The following transitions were monitored (acquisition time 150 msec/transition): (i) N,N-dimethylsphingosine (Q1/Q3, *m*/*z*, CE in parenthesis): 328.2/310.2 (26 V), 328.2/280.2 (32 V), and 328.2/110.2 (42 V) and (ii) PSY (Q1/Q3, *m*/*z*, CE in parenthesis): 462.5/444 (25 V), 462.5/282 (30 V);462.5/264 (27 V), and 462.5/252 (39 V). Transitions 328.2/310.2 (N,N-dimethylsphingosine) and 462.5/282 (PSY) were used for quantitation purposes. 

We considered mice within PND= 28 such as “early stage” (PND20-28) and mice after PND = 30 such as “late stage” (PND30-38). We used *n* = 7 mice at early stage and *n* = 12 at late stage for brain analysis, while *n* = 7 mice at early stage and *n* = 8 at late stage for optic nerves.

Brain and optic nerve lysates were used also to measure GALC activity in WT and TWI mice, as reported in [26]. Results were expressed in unit per microgram ((U/ug) = unit of enzyme per microgram of cell lysate; unit (U) = amount of enzyme that catalyzes 1 nmol of substrate per hour) and reported in percentage of the activity of the WT-early. Here, we considered mice at PND = 18–21 such as “early stage” and mice after PND = 30 such as “late stage” (PND30-34).

### 2.6. Statistical Analysis

Data are reported as average value ± the standard error of the mean (mean ± SEM), if not differently stated. All the experiments were repeated at least three times independently for each reported dataset (*n* ≥ 3 independent samples for each condition), if not differently stated. Data were statistically analyzed by GraphPad PRISM 5.00 (GraphPad Software, San Diego, CA, USA). For parametric data (the mean values obtained in each repeated experiment were assumed to be normally distributed about the true mean), Student’s *t*-test (unpaired, two-tailed) analyses were used, if not differently stated. Statistical significance refers to the results where *p* < 0.05.

## 3. Results

### 3.1. Functional Alterations in the Visual System of the Twitcher Mouse

**Figure 1:**
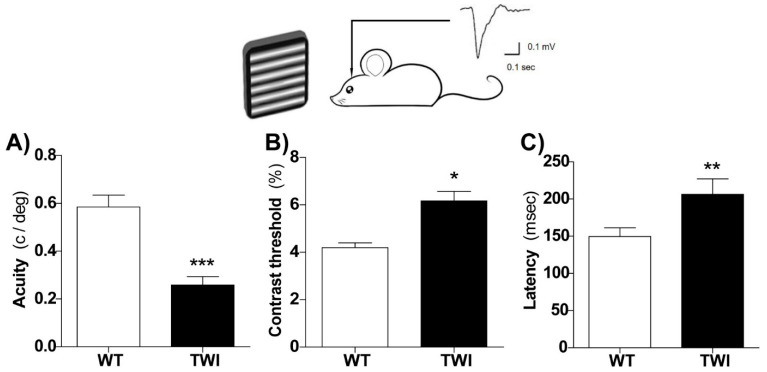
Altered functional properties of the visual cortex in Twitcher (TWI) mice. In vivo electrophysiological visual evoked potential (VEP) recordings from the primary visual cortex at postnatal day (PND)30-36 revealed reduced visual acuity ((**A**) Student’ *t*-test, *** *p* < 0.001), higher contrast threshold ((**B**) *t*-test, ** *p* < 0.01), and latency of visual responses ((**C**) *t*-test, * *p* < 0.05) in TWI mice (*n* = 7, black) compared to wild type (WT, white) control littermates (*n* = 6). Bars represent mean ± SEM.

In order to assess the functional impairments of the TWI mouse visual system, we performed visual evoked potential (VEP) recordings and measured the basic physiological parameters of the primary visual cortex. VEPs from the binocular visual cortex were recorded in vivo in PND30-P36 mice, blind to genotype at a depth of 100 or 400 µm into the cortex in response to patterned stimuli. Cortical spatial resolution (visual acuity) and contrast sensitivity, well-established measures of overall rodent as well as human visual function [37,38,39], were both impaired in the primary visual cortex of TWI mice (Figure 1). Indeed, we found a lower visual acuity (Figure 1A) and an increased contrast threshold (Figure 1B) in TWI mice in comparison to age-matched WT animals. As demyelination is expected to result in slower conduction of visual afferent input to the cortex, the VEP latency was also measured. As expected, we found that visual responses were significantly delayed in TWI mice compared to those measured in control mice (Figure 1C). VEP amplitudes were not significantly different between the two groups (data not shown). Thus, the GALC deficiency induced an immature functional visual system in TWI mice.

### 3.2. Demyelination and Gliosis in Twitcher Mice

**Figure 2:**
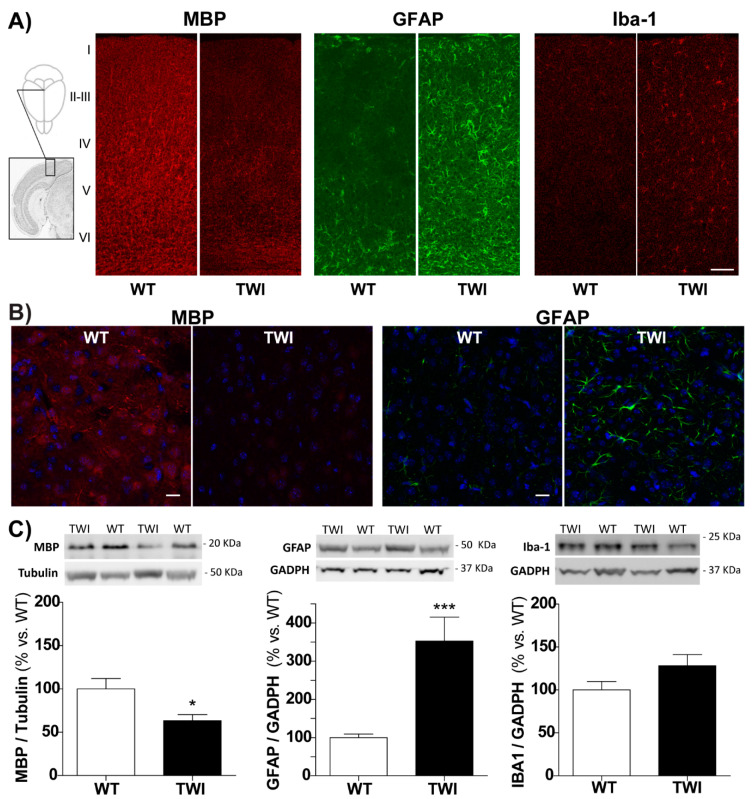
(**A**) Coronal sections through the visual cortex (inset) of representative WT (left) and TWI (right) mice (PND34) immunostained with antibodies against myelin basic protein (MBP) (marker of myelination), glial-fibrillary acidic protein (GFAP) (marker of astrogliosis), and Iba-1 (Ionized calcium-binding adaptor molecule 1, marker of microglia/macrophages). Cortical layers are indicated by Roman numbers on the left. Notice reduced myelin staining and robust gliosis in the visual cortex of TWI mice. Scale bars = 100 µm. (**B**) Representative confocal images of WT (left) and TWI (right) mouse brain (i.e., motor cortex) immunostained for MBP (marker of myelination, in red) and GFAP (marker of astrogliosis, in green), together with nuclei (DAPI, in blue): the overall degeneration status is comparable with the one of visual cortex. Scale bars = 20 µm. (**C**) Representative Western blot panels and blot analysis of MBP, GFAP, and Iba-1 levels in brain tissue lysates from WT (white) and TWI (black columns) mice. Results (normalized to Tubulin or GAPDH, Glyceraldehyde-3-phosphate Dehydrogenase, levels) were reported in % with respect to WT levels. */*** *p* < 0.05/0.001 WT vs. TWI; Student’s *t*-test. Data= mean ± SEM, *n* ≥ 18; WT mice: age range PND29-43; TWI mice: age range PND30-40.

In order to investigate the relationship between visual functional dysfunction and structural alterations, we analyzed the anatomical features of the mouse visual cortex. We qualitatively assessed the levels of myelination (myelin basic protein, MBP), astrogliosis (glial-fibrillary acidic protein, GFAP), and microglia/macrophages activation (Iba-1), which are commonly associated to neuroinflammation. At the neuroanatomical level, in the primary visual cortex of the TWI mouse (PND34), we found a very clear reduction in the myelin specific marker MBP labelling (Figure 2A, left panel) and a corresponding robust increase in the density of astrocytes and microglial cells, immunostained for GFAP and Iba-1, respectively (Figure 2A middle and right panels). 

In parallel, we found an overall reduction in MBP labelling and an increase in GFAP-positive astrocytosis in the whole TWI brain (Figure 2B), suggesting that the degeneration of the visual cortex is comparable with the degeneration present in other brain areas (e.g., motor cortex and corpus callosum). We also quantified the amount of these protein markers in total brain lysates from TWI brains by Western blot (Figure 2C). The protein markers of demyelination, astrogliosis, and microglia activation showed the same trend visualized in the TWI visual cortex. We found a significant increase in GFAP (*p* < 0.001 WT vs. TWI, Student’s *t*-test) and a reduced MBP level (*p* < 0.05 WT vs. TWI, Student’s *t*-test) in the TWI samples compared to WT littermates (Figure 2C).

Overall, demyelination and gliosis are particularly evident in the TWI visual cortex. These data demonstrate and confirm specific neuropathological alterations in the visual system of TWI mice. 

### 3.3. Psychosine Accumulation in Twitcher Tissues

**Figure 3:**
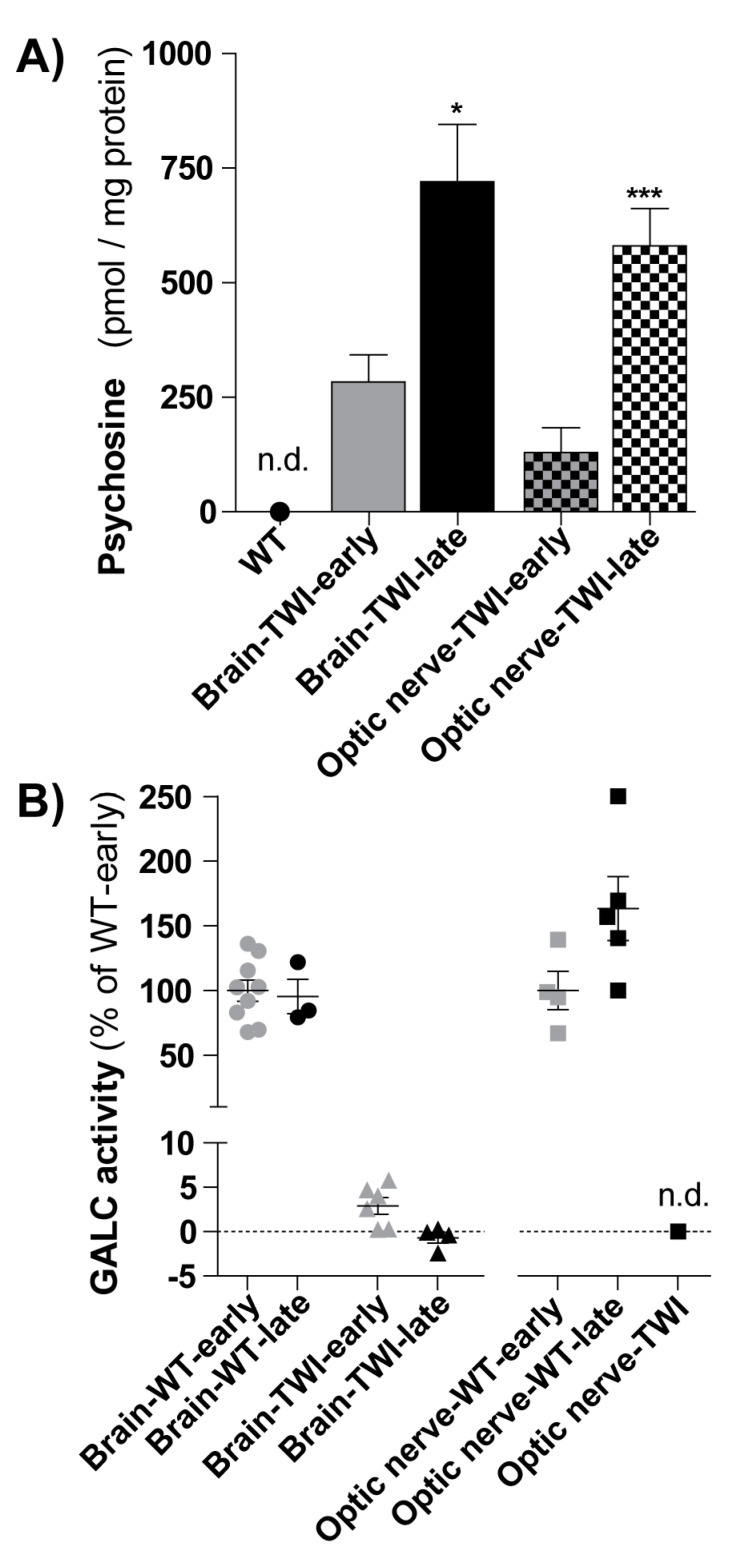
(**A**) Psychosine (PSY) quantification. PSY content was measured in brains (full-filled color columns) and optic nerves (squared columns) of TWI mice in the early stage of disease (early: PND20-28, *n* = 7) and in the late stage of the disease (late: PND30-38, *n* = 12 for brain and *n* = 8 for optic nerve). */*** *p* < 0.05/0.001 early vs. late, Student’s *t*-test. PSY was not detectable (n.d.) in WT mice, in both districts. Data = mean ± SEM. (**B**) Galactosylceramidase (GALC) quantification. GALC activity (reported in percentage in respect to the respective WT-early) was measured in brains (left; circles and triangles) and optic nerves (right; squares) of WT and TWI mice in the early stage of disease (early, in grey: PND20-21 for brains, PDN18 for optic nerves) and in the late stage of the disease (late, in black: PND30-38). GALC was not detectable (n.d.) in the optic nerves of TWI mice. Each point represents a mouse; data= mean ± SEM.

We successfully setup an analytical method for the quantitation of PSY in mouse tissue extracts [5,26]. We used a LC-ESI-tandem-MS method showing excellent sensitivity and specificity. Detection and quantitation limits were 5.2 and 8.3 ng PSY/mg protein (11.3 and 18.0 pmol/mg protein), respectively. These values allowed us to quantify PSY levels in the brain and optic nerve of TWI mice at different disease-time progression (Figure 3A).

We found that PSY accumulated with age progression in both the brain and optic nerve of TWI mice. In details, in the brain tissue, PSY levels increased from 284 ± 60 pmol/mg protein in TWI mice at early stage of the disease (within PND28) to 720 ± 130 pmol/mg protein at late stage (from PND30) (*p* < 0.05, Student’ *t*-test). Similarly, in optic nerves, PSY levels increased from 130 ± 50 pmol/mg protein in TWI at early stage to 580 ± 80 pmol/mg protein at late stage of disease (*p* < 0.001, Student’ *t*-test). As comparison, the PSY level in the correspondent WT tissues was not detectable in the same analysis conditions. In parallel, we quantified also GALC activity (Figure 3B). In both the brain and the optic nerve, GALC activity did not changed with age progression in WT mice, while it was, as expected, negligible in TWI mice (Figure 3B).

These data show that PSY accumulated in the brain and optic nerves of TWI mice already at early stages of the disease and further increased with time progression. PSY is a characteristic hallmark of KD, also at the level of visual system.

## 4. Discussion

In the present work, we analyzed the visual system functionality and the myelination status of visual cortical neurons in the Twitcher mouse, as a model of Krabbe disease. The in vivo electrophysiological recordings showed defective basic functional properties of the TWI primary visual cortex. In particular, we found a reduced visual acuity as well as contrast sensitivity and a delayed visual response. Specific neuropathological alterations were present in the visual cortex of the TWI mouse. We found reduced myelination, astrogliosis, and microglia activation, as showed by the biomarkers for myelin (MBP), astrocytes (GFAP), and microglia/macrophages (Iba-1), respectively, by both immunostaining and Western blot. Moreover, we quantified the evolution of the PSY accumulation in brain and optic nerves by high-pressure liquid chromatography-mass spectrometry methods. PSY, the characteristic hallmark of KD, started accumulating already at the early stage of the disease, both at brain and optic nerve level, and increased with time in both districts.

To the best of our knowledge, this study provides the first report about functional and structural defects of the visual system in the TWI mouse. Only recently, VEPs have been investigated in KD patients, with the first evidence in literature in 2000. It has been found that VEPs are abnormal in KD children and VEPS have been suggested as helpful early sentinel signs and tests for the objective evaluation of KD patients [22,40]. Here, for the first time, we found VEPs abnormalities also in TWI mice: the increased latency registered in TWI mice is in line with the neuronal demyelination that we observed histologically in the visual cortex and that leads to a slower signal conductance.

We here further suggest that visual acuity and contrast sensitivity might also be impaired in KD. In fact, we found that these features are deteriorated in TWI mouse primary visual cortex, likely because the visual cortex has not developed properly. For a complete evaluation of KD patients and as a diagnostic marker, the acuity and contrast sensitivity measurements could also be exploited in the clinic. In support of this, the usefulness of visual response analysis for the monitoring of functional alterations in children with severe disabilities was recently described in a study where abnormal VEPs and visual acuity deterioration were identified as biomarkers for another pediatric disease, the Rett Syndrome [41]. Noteworthy visual acuity as well contrast sensitivity can be easily assessed in children with various methods [37], including behavioral ones (electrophysiological that are technically more difficult to implement in a child).

At structural level, we confirmed demyelination as a typical neuroanatomical hallmark of the KD CNS. In addition, we found astrogliosis and microglia activation in the TWI visual cortex and whole brain, in agreement with the literature and the inflammatory profile of KD [8].

Little is known regarding the molecular progression of KD. The most obvious candidate as a molecular catalyst for KD is psychosine, even if its mechanism of action remains elusive. Here, PSY levels are increased in both the brain and the optic nerve of TWI mice. We found that PSY accumulation correlates with the reduced myelination in TWI mouse CNS and this is in line with the causative effect of PSY on oligodendrocytes death that it has been suggested in literature [7]. Thus, the visual functional defects we observed in TWI mice visual cortex might be due to the increased levels of PSY, at least concerning the visual response latency. At the best of our knowledge, we did not find any measurements about the PSY levels in TWI optic nerves, although its accumulation was already proposed as possible cause of the optic nerve structural anomaly in a human KD patient in 1978 [42]. It has been recently reported that neuronal GALC is required for proper brainstem development and that the deficiency of GALC is critical for KD pathogenesis [43]. In parallel to psychosine analysis, we quantified also GALC activity in the brain and optic nerves, GALC activity did not changed with age progression in WT mice, while it was, as expected, negligible or not detectable in TWI mice.

Overall, our results suggest that the visual system is an optimal and accessible model for studying KD. Moreover, our results may have important implications for KD research since we identified visual system parameters useful for the preclinical evaluations of new therapeutic strategies in the TWI mouse.

For example, recent studies suggest that gene therapy might be a valid option for KD treatment and gene technology constantly develops [13]. The visual system might be exploited as the preferential field of investigation to examine the impact of gene therapy even that of last generation, on brain defects due to KD. In particular, it might be useful to establish the adequate dose of gene therapy, able to completely correct KD impairments. In support of this, a recent study showed that low but not high-dose gene therapy-treated dogs had visual system deficits [44].

Importantly, visual defects have been similarly reported also in animal models of other LSDs. In a mouse model of Cystinosis (CTSN), for example, an LSD characterized by abnormal accumulation of cystine, the eye is one of the first organs affected, with visual impairment in the second decade of life. The tempo spatial pattern of cystine accumulation in CTSN mice parallels that of patients, validating CTSN mice as a model for the visual anomalies of CTSN and for the testing of novel ocular cystine-depleting therapies [28,29]. Visual abnormality has also been well characterized in model of Sandhoff (SD) and GM1 gangliosidosis mice. Although electroretinograms appeared normal in the SD and the GM1 mice, VEP were subnormal in both these mutants, indicating clear visual impairments [30]. Additionally, visual system degeneration and impairment have been demonstrated also in a mouse model for Mucopolysaccharidosis type IIIA (MPS-IIIA, Sanfilippo A), a severe LSD caused by the inherited deficiency of sulfamidase, and in parallel in MPS-IIIA patients [31].

The importance to acquire a deep knowledge about the visual system of LSD mouse models with visual impairment is further supported by the fact that the acquired knowledge can be further exploited for monitoring human patients. In this regard, the visual system might represent a new target for monitoring the effect of the currently standard of care for presymptomatic patients, the HSCT, that has been shown to effectively treat the CNS but not the PNS [13]. In addition, the importance of visual system studies in KD disease is highlighted by the fact that the eye can be also used as an administration route for testing experimental therapies [45].

Overall, these results constitute the first complete characterization of the TWI visual systems. Our data set a baseline for an easy testing of potential therapies for this district, which is also dramatically affected in KD patients. The new knowledge might also be further translated in human patients to shorten the KD diagnosis process.

## Data Availability

Data is contained within the article.
